# Medical decision-making competence regarding puberty suppression: perceptions of transgender adolescents, their parents and clinicians

**DOI:** 10.1007/s00787-022-02076-6

**Published:** 2022-09-17

**Authors:** Lieke Josephina Jeanne Johanna Vrouenraets, Annelou L. C. de Vries, Marijn Arnoldussen, Sabine E. Hannema, Ramón J. L. Lindauer, Martine C. de Vries, Irma M. Hein

**Affiliations:** 1 Department of Medical Psychology, Willem Alexander Children’s Hospital, Leiden University Medical Center, Leiden, The Netherlands; 2 Department of Medical Ethics and Health Law, Leiden University Medical Center, Albinusdreef 2, 2333 ZA Leiden, The Netherlands; 3 Department of Child and Adolescent Psychiatry, Emma Children’s Hospital, Amsterdam University Medical Centers, Location VUmc, Amsterdam, The Netherlands; 4 Center of Expertise on Gender Dysphoria, Amsterdam University Medical Centers, Location VUmc, Amsterdam, The Netherlands; 5 Department of Paediatric Endocrinology, Amsterdam University Medical Centers, Location VUmc, Amsterdam, The Netherlands; 6 Department of Paediatrics, Leiden University Medical Center, Leiden, The Netherlands; 7 Department of Child and Adolescent Psychiatry, Amsterdam University Medical Centers, Location AMC, University of Amsterdam and Levvel, Amsterdam, The Netherlands

**Keywords:** Adolescents, Gender dysphoria, Gender incongruence, Puberty suppression, Medical decision-making competence, Qualitative study

## Abstract

According to international transgender care guidelines, transgender adolescents should have medical decision-making competence (MDC) to start puberty suppression (PS) and halt endogenous pubertal development. However, MDC is a debated concept in adolescent transgender care and little is known about the transgender adolescents’, their parents’, and clinicians’ perspectives on this. Increasing our understanding of these perspectives can improve transgender adolescent care. A qualitative interview study with adolescents attending two Dutch gender identity clinics (eight transgender adolescents who proceeded to gender-affirming hormones after PS, and six adolescents who discontinued PS) and 12 of their parents, and focus groups with ten clinicians was conducted. From thematic analysis, three themes emerged regarding transgender adolescents’ MDC to start PS: (1) challenges when assessing MDC, (2) aspects that are considered when assessing MDC, and (3) MDC’s relevance. The four criteria one needs to fulfill to have MDC—understanding, appreciating, reasoning, communicating a choice—were all, to a greater or lesser extent, mentioned by most participants, just as MDC being relative to a specific decision and context. Interestingly, most adolescents, parents and clinicians find understanding and appreciating PS and its consequences important for MDC. Nevertheless, most state that the adolescents did not fully understand and appreciate PS and its consequences, but were nonetheless able to decide about PS. Parents’ support of their child was considered essential in the decision-making process. Clinicians find MDC difficult to assess and put into practice in a uniform way. Dissemination of knowledge about MDC to start PS would help to adequately support adolescents, parents and clinicians in the decision-making process.

## Introduction

The World Professional Association for Transgender Health (WPATH) Standards of Care (7th version) and the Endocrine Society Guideline on transgender care for children and adolescents recommend treatment with puberty suppression (PS; using Gonadotropin-Releasing Hormone (GnRH) agonists) provided that certain criteria are fulfilled^1^ (see for a complete overview of all diagnostic criteria for PS Table 1) [1, 2]. It is recommended to start PS when, among other criteria, adolescents suffer from an intense and long-lasting pattern of gender dysphoria/gender incongruence and after they first exhibit physical changes of puberty (at least Tanner stage 2). The aim of using PS in this context is to suppress, in a reversible manner, further development of secondary sex characteristics to allow the adolescent more time and rest to explore their gender before decisions are made on gender-affirming hormones (GAH) with more irreversible effects. Besides, it prevents psychological distress associated with the undesired endogenous pubertal development, as several adolescents stated in an interview study regarding the function of PS [3]. In addition, the physical outcome may be more satisfactory when using PS in the early stages of puberty because some surgeries, such as mastectomy, may then not be necessary or less invasive (i.e. periareolar rather than inframammary approach) because the development of secondary sex characteristics is prevented [4]. Currently, the evidence base for the positive implications of treatment is still limited and treatment teams applying PS may experience feelings of unease [5]. Their concerns regard the lack of data on its impact on physical, psychosocial, and cognitive development in the long-term, and the consequences for fertility [6, 7]. In an interview study, clinicians report worries about the risk of regret and the lack of long-term data on possible side effects of PS [5]. Transgender adolescents themselves express some hesitations to start treatment with PS too, e.g. about the ability of adolescents to make informed decisions regarding medical treatment at the age of 12 or younger [8]. Research shows that transgender people, after sex reassignment, have significantly higher risks for suicidal behaviour, psychiatric morbidity, and mortality compared to the general population [9, 10]. Nevertheless, it is unknown whether these results are the same for transgender people who started treatment with PS in the early stages of their puberty. Besides, it is good to keep in mind that these studies do not tell us anything about the exact causes of these increased risks; some of the outcomes might be related to, for example, transgender people’s experiences of living in a discriminatory and rejecting society (i.e. minority stress) instead of solely being related to post-surgical outcomes [e.g. 11].

**Table 1:** Diagnostic criteria for treatment with puberty suppression for adolescents

Adolescents are eligible for treatment with puberty suppression if:
The adolescent has demonstrated an intense and long-lasting pattern of gender dysphoria or gender nonconformity (whether expressed or suppressed)
The gender dysphoria emerged or worsened with puberty’s onset
Any concurrent psychological, social, and/or medical issues that could interfere with the treatment (for example, that may compromise compliance with the treatment) have been addressed, such that the functioning and situation of the adolescent are stable enough to start the treatment
The adolescent is having sufficient mental capacity to give informed consent to the treatment
The adolescent and/or parent(s)/other caretaker(s) (depending on the adolescent’s age and local laws) has/have given informed consent after being informed about the effects of the treatment and fertility preservation options
The parent(s)/other caretaker(s) is/are involved and supporting the adolescent throughout the treatment process
A pediatric endocrinologist or other clinician with experience in the assessment of puberty agrees with the indication of the health care provider to start puberty suppressing treatment
The adolescent’s puberty has started (Tanner stage ≥ G2/B2)
The adolescent has no medical contraindications to treatment with puberty suppression

Adolescents may present or be diagnosed with gender dysphoria during or after the completion of endogenous puberty and may therefore start PS at various stages of pubertal development. Most adolescents who start treatment with PS subsequently start treatment with GAH and surgery afterwards [12]. Some adolescents discontinue their PS treatment. Of the latter group, most no longer wish gender-affirming treatment, while some commence treatment with GAH and/or surgery later in life, such as in adulthood [12]. Besides, providing solely psychological support, and see if adolescents can accept themselves without any medical intervention, is always considered when working according to the international guidelines too [1, 2]. Research shows that about 22 percent of the youth referred to a Dutch specialized gender identity clinic do not start affirmative medical treatment, being PS and/or GAH [13].

As far as currently known, the effects of PS on the development of secondary sex characteristics and gonadal function are reversible when discontinued [2]. Nevertheless, transgender adolescents who start PS at a young age and subsequently start treatment with GAH and undergo a gonadectomy, may not be able to pursue fertility preservation since these adolescents never undergo their endogenous puberty [14–16]. On the other hand, one should keep in mind that refraining from PS could be harmful as well, with potential life-long psychological, medical, and social consequences, such as personal physical discomfort, stigmatization, and difficulties with social integration and social function [17–20]. So, these young adolescents make decisions that may have life-long consequences. Even though it is recommended to involve parents when adolescents decide about starting PS, the issue whether these adolescents are capable of making these decisions is an important one [1, 21]. According to international guidelines, one of the criteria for treatment with PS is that adolescents are competent to give informed consent [1, 2]. However, in society, there is doubt about this competence [e.g. 16, 22–27]. Furthermore, both transgender adolescents themselves and clinicians mention medical decision-making competence (MDC) as one of the main topics in the debate regarding treatment with PS [5, 8, 28].

MDC describes the capacities that a person needs to make an autonomous medical decision [29]. To have MDC, one needs to fulfill the following four criteria: (1) understanding the information relevant to one’s condition and the proposed treatment; (2) appreciating the information and relating it to one’s circumstances including one’s current medical situation and one’s values; (3) reasoning about benefits and potential risks of the options; and (4) communicating a choice [30]. MDC is relative to a specific decision and context. It is one of the three prerequisites to give valid informed consent, besides being well-informed and without coercion [31, 32].

In December 2020, the High Court of Justice in London ruled that transgender youth under the age of 16 are highly unlikely to fully understand the long-term effects of PS, and therefore are not competent to decide on treatment with PS [33]. As a result of this verdict transgender adolescents in England could no longer start PS before age 16 unless a court order was obtained [34]. However, in September 2021 the Court of Appeal overturned the High Court’s ruling of December 2020 [35]. Furthermore, in Sweden pediatric endocrinologists stopped providing PS to newly referred transgender adolescents in May 2021 because of, among others, concerns regarding harmful long-term consequences [36]. In summary, adolescents’ MDC to start PS is and has been under discussion for some time among both advocates and opponents of the use of PS in transgender adolescents [e.g. 17, 26, 37–39].

Adolescents’ MDC has often proved difficult to assess and is usually evaluated implicitly in clinical settings [40]. Additionally, there is little empirical evidence on transgender adolescents’ competence to decide on PS. To our knowledge, there is only one study, from the Netherlands performed in our centers, that has examined this by a structured replicable interview. That study shows that the vast majority (89%) of transgender adolescents (aged 10–18 years) about to start PS treatment are competent to consent to this treatment [41].

Little research has examined the ideas and considerations of adolescents themselves and their parents regarding adolescents’ MDC to start PS. An interview study showed that clinicians stated that they find it important that the adolescents mature a little further during the period they receive PS so that they will be better able to decide about proceeding to GAH and carefully consider their decision’s consequences. This implies that these clinicians assume that the adolescents, when they decide on PS, are not always competent yet to decide on GAH [3]. Insight into the stakeholders’ perceptions of adolescents’ MDC will help to further improve care and support for adolescents in their decision-making process. Therefore, we performed an interview and focus group study to investigate the perceptions of transgender adolescents who proceeded to GAH after PS, adolescents who discontinued treatment with PS, their parents, and clinicians regarding transgender adolescents’ MDC concerning PS.

## Methods

### Participants

The interviews and focus groups were conducted in the context of a larger study on transgender adolescents’ competence to consent to PS and the function of this treatment. Study methods are described in full in the article about the perceptions of the various informants on the function of PS [3]. Briefly, transgender adolescents who proceeded to GAH after treatment with PS (‘continuers’), adolescents who discontinued treatment with PS (‘discontinuers’), and their parents were recruited from the gender identity clinics in Amsterdam and Leiden between January and September 2019. The informants were interviewed using a topic list (see Appendix A) to explore their considerations and experiences. The same topics were discussed in focus groups with clinicians of the two Dutch gender teams.

Semi-structured individual interviews were conducted with 14 adolescents and 12 parents. The informants consisted of:

1. Eight transgender adolescents who were treated with PS and subsequently with GAH;

2. Six adolescents who had been treated with PS and had discontinued this treatment;

3. Eight parents of adolescents who were treated with PS and subsequently with GAH;

4. Four parents of adolescents who had discontinued treatment with PS.

Inclusion criteria for the adolescents who had continued treatment (group 1) were: (a) diagnosis of gender dysphoria according to DSM-IV or DSM-5, depending on which version of the DSM was used at the time of diagnosis [42], (b) had started PS at age 10–15 years, (c) had used PS for at least 12 months, (d) had used GAH for at least six months, and (e) age at the time of the interview between 15 and 20 years. The aim was to have at least as many adolescents in group 1 as in group 2. Therefore, thirteen consecutive adolescents were asked to participate when they attended their regular follow-up appointment. Eight adolescents agreed to participate. Five adolescents declined for various reasons.

Inclusion criteria for the adolescents who had discontinued treatment (group 2) were: (a) diagnosis of gender dysphoria according to DSM-IV or DSM-5, depending on which version of the DSM was used at the time of diagnosis [42], (b) had started PS at age 10–17 years (a wider age range was chosen for those that discontinued PS to allow the inclusion of as many participants as possible given the limited number of individuals that discontinued PS), and (c) had discontinued PS treatment. Out of 1015 adolescents diagnosed with gender dysphoria between 2000 and 2018 at the Amsterdam or Leiden gender identity clinic, twenty adolescents in total were eligible. Eight adolescents could not be reached, mostly because their contact details were no longer up to date. One was not contacted because he had previously indicated that he did not want to be approached for research purposes. Two adolescents were not contacted because their clinician thought this was inappropriate due to, among others, comorbid mental health difficulties. Nine adolescents were asked to participate. Two adolescents declined without giving a reason, one parent did not want her child to participate because she did think that was not in the child’s best interest, and six adolescents agreed to participate. Characteristics of the two groups of adolescents are presented in Table 2.

**Table 2:** Characteristics of participating adolescents

Variables	Adolescents who discontinued treatment	Adolescents who continued treatment
*N*	6	8
Birth assigned girls	5^a^	4
Birth assigned boys	1^b^	4
Age during interview (median; range) (years)	17.5; 14–27	17.9; 15–18
Age when visiting gender identity clinic for the first time (median; range) (years)	14.3; 11–15	11.3; 10–13
Age start PS (median; range) (years)	15.2; 12–17	12.3; 10–14
Duration of PS (median; range) (months)	10; 1–14	35; 21–48
Duration diagnostic trajectory before starting PS treatment (median; range) (months)^c^	10; 6–22	9; 6–12
Full-Scale IQ (median; range)	100; 98–124	104; 76–132

^a^Two adolescents identified as transboy, one as a-gender, one as genderfluid, and one as cis-gender girl at the time of the interview

^b^Gender identity at the time of the interview: a-gender

^c^Diagnostic trajectory before starting PS treatment; In the Netherlands, transgender adolescents undergo a diagnostic trajectory, consisting of psycho-diagnostic assessment and several sessions with a mental health provider over a longer period of time, when assessing eligibility for PS

The parents of all interviewed adolescents who continued treatment were asked to participate (group 3). Eight parents (seven biological mothers and one biological father) agreed. Four parents (group 4; three biological mothers and one adoptive mother) of adolescents who had discontinued treatment were asked to participate in the study and all agreed. The other parents were not asked because of logistic reasons (e.g. they could not be reached by phone in time prior to the appointment).

In addition, two focus groups with clinicians working at the two treatment teams were held. The informants were purposefully selected based on their discipline (all different disciplines working within both teams participated to assure representativeness for the complete treatment team; i.e. three child and adolescent psychiatrists, four child and adolescent psychologists and three pediatric endocrinologists).

### Procedure

Two authors of this study conducted the interviews. Both had interview experience and worked as a clinician at one of the gender identity clinics (MA and LV). They had not been involved in the diagnostic assessments of the adolescents they interviewed. Initial interview questions were formulated after review of the relevant literature and discussion within the research team involving all authors. The interview guide contained no close-ended questions (see Appendix A).

One of the authors (MV) facilitated the two focus groups. During the focus groups the questions asked in the interviews were presented along with several anonymous quotes from the interviews to get the conversation started. The participants were asked whether they agreed with and/or identified with the quotes. Furthermore, the participants were invited to express possible other views they held on these topics.

All interviews and focus groups were conducted in Dutch, and were audio-taped and transcribed verbatim. Written informed consent for participation and tape recording was obtained before each interview and each focus group. The study was approved by the institutional review board of the Amsterdam University Medical Centers, location VUmc, and the Leiden University Medical Center.

### Analysis

Data analysis was based on hermeneutic analysis [43, 44]. After an initial open reading of the data, two of the authors presented some preliminary (sub)themes (MA and LV). Besides, one of these authors analyzed the transcripts by selecting representative quotations for each of the defined themes, taking care to draw quotations from all data sources. Then, the same two authors conducted an additional round of analyses to assess whether the (sub)themes enabled them to accurately subdivide the outcome of the data. They also re-analyzed the transcripts to select representative quotations. Then, through a deliberative process, the authors redefined the initial (sub)themes until they reached a consensus. The quotations were initially translated from Dutch into English by one of the authors (LV). The other authors, who are all bilingual, checked, and if necessary, revised these translations (they were also provided with the original Dutch quotations).

## Results

From the interviews and focus groups, 10 themes emerged regarding transgender adolescents’ MDC to start PS. These 10 themes can be merged into three main themes: (1) challenges when assessing MDC to start PS, (2) aspects that are considered when assessing the adolescent’s MDC, and (3) relevance of MDC. Representative quotations are presented to illustrate the themes identified.

### Challenges when assessing MDC to start PS

During the interviews and focus groups the informants mentioned several aspects that challenged the assessment of MDC to start PS. Six subtopics further emerged from the data.

#### Understanding and appreciating consequences of PS

Most adolescents and parents mentioned that certain aspects of (the impact of) the treatment simply cannot be understood and appreciated by adolescents below a certain age.

*I think I had thought about it [starting treatment with puberty suppression or not starting this treatment] pretty well. But as a 12 or 13 year-old, you can’t really judge what it’s all about. So I had thought about it [starting the treatment or not], but only as much as I was able to at the time [I decided to start with the treatment]* (Interview with a transgirl who continued PS; age at start PS: 12.9; age at interview: 17.8)

*It doesn’t really mean much to a 12 year-old when you’re talking about osteoporosis. She [my daughter] understood [what osteoporosis meant], but she thought 'what does it matter’, we’ll see about that later* (Interview with a parent of a transgirl who continued PS; age at start PS: 12.9; age at interview: 17.8)

Some adolescents, both continuers and discontinuers, wondered whether they were able to understand and appreciate the consequence of possible loss of fertility if they were to proceed to GAH and possibly gonadectomy, and whether they were able to carefully consider the possibility to freeze sperm or store oocytes before they started the treatment with PS. Some adolescents stated that during the period they were treated with PS, they started to realize what the impact of some consequences could be. Worth mentioning is that one parent whose child froze sperm mentioned the impact which the process of fertility preservation had on her child and on herself, instead of the impact of the possible loss of fertility.

*The first few months I was very happy with it [the inhibition of secondary sexual characteristics] until I realized what if I won’t be fertile anymore* (Interview with an assigned female at birth who had discontinued PS; age at start PS: 17.0; age at discontinuation PS: 17.9; age at interview: 27.8)

*At the moment I know that I would like to have children when I grow older [while at the time I made the decision regarding starting treatment with puberty suppression, I did not have a desire to have children] [...] That’s the only thing I wonder about, whether I was able enough to make that decision at the time* (Interview with an assigned female at birth who had discontinued PS; age at start PS: 17.0; age at discontinuation PS: 17.9; age at interview: 27.8)

*Before she [my daughter] started treatment with puberty suppression, she had frozen sperm. I found that very intense. Ehm... for her too of course. [...] I thought it had quite an impact on such a young child, who had to go into that room to fill up a jar [with sperm]. […] I found that quite difficult to deal with [as a parent]* (Interview with a parent of a transgirl who continued PS; age at start PS: 14.2; age at interview: 17.9)

The clinicians mentioned several consequences of PS which give them a feeling of unease when treating adolescents with PS. One of these consequences, a concern that all clinicians shared and which was mentioned by adolescents and parents too, is the possible loss of fertility if adolescents proceed to GAH and possibly gonadectomy.

*I think that the part regarding wanting to have children is a tricky one. They [the adolescents] just can’t understand and appreciate that [the impact of possible infertility]* (Focus group with clinicians)

The clinicians, just as most adolescents and parents, stated that not being able to understand and appreciate the impact of certain consequences of PS is inherent to the adolescent’s age and/or developmental stage, for example, the possible consequence of loss of fertility for one’s future life and relationships. Furthermore, they stated that even some adults are unable to understand and appreciate the impact of such consequences.

*How can you leave such a choice [whether or not you want biologically related children when you are older] to these children?* (Focus group with clinicians)

*That [possible infertility due to treatment with puberty suppression and subsequent gender affirming hormones and surgery] is a very complicated one. As if children of that age [12 or 13 years old] can even begin to imagine what it [infertility] really implies* (Focus group with clinicians)

Apart from the possible loss of fertility, adolescents, both continuers and discontinuers, parents, and clinicians mentioned other consequences of PS that are difficult to understand and appreciate for adolescents prior to the start of treatment. For example, several adolescents mentioned that they hadn’t realized their peers would undergo pubertal development while they stood ‘still’ as their puberty was suppressed and that they found this difficult to cope with. This had a negative psychological impact.

*I did have the feeling that I stood still while the rest [my peers who did go through pubertal development] went on [...] [Before starting the treatment with puberty suppression] I hadn’t thought very much about what that could do to you mentally. [...] I was quite depressed during that time. And [...] I think that it [the fact that I had the feeling I stood still while my peers went through their pubertal development] also played a part in how I felt [depressed] at the time. I had not foreseen that beforehand* (Interview with a transgirl who continued PS; age at start PS: 12.0; age at interview: 18.1)

Additionally, some clinicians stated that they think that it is difficult for an obese adolescent to understand and appreciate the impact of not being eligible to undergo certain surgeries following PS and GAH if they were to remain obese.^2^

#### Uncertainties regarding the long-term effects of PS

Some parents stated that they themselves knew little about the consequences of the treatment. One parent indicated that it is a challenge that no one can ever tell what the outcome would have been without medical treatment. Furthermore, some parents stated that they themselves would never take medication with unknown long-term effects.

*[Starting the treatment with puberty suppression was] a bit scary for all of us in the sense that we didn’t know whether we were doing the right thing or whether we were going to stuff our child full of things of which you do not know the consequences yet* (Interview with a parent of a transboy who continued PS; age at start PS: 10.9; age at interview: 17.6)

*What I think is difficult about puberty suppression is that you don’t know exactly what you’re suppressing; you don’t know what she [my daughter] would have become if she hadn’t used that [treatment with pubertal suppression] [...] you don’t know what you're inhibiting. [...] Yes, you [know that you] suppress puberty, but you don’t know what it [my daughter’s puberty] would have looked like [without the treatment with puberty suppression]* (Interview with a parent of a transgirl who continued PS; age at start PS: 12.4; age at interview: 18.6)

*You would never do that to yourself; you would never inject yourself with something you don’t know the long-term consequences of* (Interview with a parent of a transgirl who continued PS; age at start PS: 12.4; age at interview: 18.6)

Clinicians stated that it is difficult to inform adolescents and their parents about possible consequences of PS which are not yet known. In addition, they mentioned that no one can foresee what impact certain consequences will have on the quality of life of the adolescent.

*You can’t properly inform people about what is not known [regarding (possible consequences of) the treatment], except that there are uncertainties. That’s very difficult* (Focus group with clinicians)

*Of course, you don’t exactly know that [what the consequences of treatment with puberty suppression may be]. And you don’t exactly know what effect that [those consequences] will have on the person’s well-being later in life either* (Focus group with clinicians)

#### The parents’ role, influence and responsibility

Some adolescents, both continuers and discontinuers, and parents mentioned the substantial role some parents play in the diagnostic trajectory and decision-making, whereas other adolescents were not sure to what extent their parents had weighed the pros and cons of the treatment.

*My parents mostly investigated it [what treatment with puberty suppression entails] for me, because I really didn’t want to know anything about it […] I just couldn’t talk about it and I didn’t want to look anything up [regarding the treatment] because doing so reminded me of being [a] transgender [person]. But because my parents are like that, I ended up where I am now. Otherwise, it would have been a different story* (Interview with a transgirl who continued PS; age at start PS: 12.0; age at interview: 18.1)

*Yes, we as parents and I [the mother] in particular [have weighed the pros and cons of the treatment]. [...] I like to know what to expect, so I read up on things a bit more. My son isn’t like that; he hears it [the possibility to start the treatment], accepts it, and goes on* (Interview with a parent of a transboy who continued PS; age at start PS: 11.9; age at interview: 18.5)

One adolescent mentioned that his parents were involved and supported him in the decision-making process, but that the decision whether or not to start the treatment was made entirely by himself.

*It was entirely my own choice [to start treatment with puberty suppression]. My father and mother had virtually nothing to do with it. Yeah, of course they were there for support and things like that, but the choice was really my own* (Interview with a transboy who continued PS; age at start PS: 12.0; age at interview: 16.3)

All clinicians stated that most children and adolescents need support when going through the decision-making process, from either their parents or clinicians, and that parents and/or clinicians have an important role in the decision-making.

*The point is that you [the adolescent deciding about puberty suppressing treatment] are then at an age at which you cannot yet understand and appreciate [the consequences of the treatment], and [that you as a health care provider] basically make a choice for them [the adolescents]* (Focus group with clinicians)

Clinicians mentioned the role and sometimes strong influence parents can have in the diagnostic process and in decision-making.

*It is more the anticipated fear or agony of the parents. In these cases, I sometimes feel that the parents are urging to start the treatment [with puberty suppression] more than the adolescents themselves, because they [the adolescents] are not yet so concerned with the puberty suppression, and whether or not it [starting this treatment] is possible. Sometimes it’s difficult when I have the feeling that the parents are very much in a hurry and ‘pushing’* (Focus group with clinicians)

*I think that parents are very influential in that. How do parents talk about it [the treatment with puberty suppression]? How do they talk to each other [parents together]? I think that at that age [when the child is 10 or 11] that is very closely connected to how the child thinks about treatment* (Focus group with clinicians)

The clinicians stated that in some of these cases they find it hard to distinguish between the adolescent’s wishes and the parents’ suffering or anticipated fear.

*On the other hand, it is very complicated [...] when parents have already started that [social] transition against the advice [of the health care providers] and when they are so on top of it [starting the treatment with puberty suppression], that you wonder to what extent the child’s agony is his/her own* (Focus group with clinicians)

*Aren’t we [clinicians] reading into it, or aren’t parents reading into it? Where are these signals coming from? Are they [these signals] coming from the adolescents themselves or are they coming from the people around them? Are they [the adolescents] being colored [by the thoughts and opinions of the people around them]? These are all pretty complicated things* (Focus group with clinicians)

Additionally, the clinicians asked themselves what role parents and clinicians should have in the decision-making process. Who is responsible for the decision and its consequences? Some stated that, on the one hand, parents are responsible, since they are the ones giving informed consent according to the law (for adolescents < 12 years of age; for those aged 12–16 together with the adolescents). On the other hand, some clinicians wondered how parents can make a decision based on the interpretation of the feelings and behavior of their child. Furthermore, they pointed out the large role of clinicians in assessing which adolescents would benefit from treatment.

*I feel with those young children [about 11 years old] that the parents take over the medical decision-making competence from the children. [...] Legally that’s also the case; they [the parents] decide for the child* (Focus group with clinicians)

*Do we [the health care providers] consider ourselves most competent in medical decision-making in the whole process [diagnostic trajectory] of gender dysphoria? Considering all people involved, who are most competent in medical decision-making? Especially when you think about the fact that we [the health care providers] have such an important role in decisions about the treatment. Does that then imply, that we consider ourselves most competent to understand and appreciate what is best for the adolescent [whether or not to start treatment with puberty suppression]?* (Focus group with clinicians)

#### Choosing between two negatives; is there really any choice?

Most adolescents who proceeded to GAH after PS and their parents stated that they did not feel they had a choice whether or not to start PS. Strikingly, none of the adolescents who discontinued PS or their parents explicitly stated that they had the feeling that they did not have a choice whether or not to start PS.

*For me [...] it was never really a choice. [...] Of course it's a choice in the way that you can choose to do it [start treatment with puberty suppression or not], but in my mind it was never really a choice, but just something I wanted to do to move forward in the journey* (Interview with a transgirl who continued PS; age at start PS: 12.4; age at interview: 18.5)

*We [the parents] said to each other, we would not have let her [our daughter] make any other radical decision at the age of twelve. If she had said ‘well I really don’t need any more schooling’ at the age of twelve [...] [then we would have said] that’s out of the question, because we are your parents and we decide that you do have to go to school [...] You feel like you don’t have a choice [about whether or not to start treatment with puberty suppression]. I’m glad it [this treatment] is available, but we didn’t really experience it as having any choice. [...] My husband and I felt we had to choose between two evils and concluded we’d better choose the puberty suppressing treatment. [...] Of course it’s sad that she might die sooner because of all the chemicals that she has to take, but on the other hand there’s no point in living your life if you’re not able to be yourself* (Interview with a parent of a transgirl who continued PS; age at start PS: 12.4; age at interview: 18.6)

On the other hand, many adolescents, both continuers and discontinuers, and their parents mentioned that they simply accepted possible negative consequences of the treatment and did not really take them into consideration.

*At the time I didn’t think very much about the pros and cons of puberty suppression [...] I had really already made up my mind [even before I had heard about the disadvantages of the treatment]* (Interview with an assigned female at birth who had discontinued PS; age at start PS: 16.7; age at discontinuation PS: 17.0; age at interview: 19.5)

*No, because for her there were no disadvantages, only advantages [of the treatment]. [...] We [the parents and daughter] never actually talked about the disadvantages, except that the injections are annoying and that sometimes you can feel unwell [because of the injections]. But she didn’t take that into consideration. [...] The other way [not starting treatment with puberty suppression] was not an option. [...] So to what extent can one even speak of a consideration? The disadvantages are just part of the deal* (Interview with a parent of a transgirl who continued PS; age at start PS: 12.0; age at interview: 18.1)

#### Defining MDC

The term MDC per se was only discussed during the focus groups with the clinicians. Most clinicians encounter difficulties defining MDC: exactly what should an adolescent understand regarding the treatment, or what should one be able to explain to be considered competent to consent? Additionally, they wondered what the term ‘understanding’ means in this context.

*What does medical decision-making competence entail? […] You can talk about medical decision-making competence as in ‘do you know what happens when you use puberty suppressing treatment and do you know what the full medical trajectory entails’, so that you are aware that if you start treatment with puberty suppression, you will have to have surgeries in the future to get a penis. Is that what it [medical decision-making competence] entails? Or is it [medical decision-making competence] about the fact that if you use puberty suppression, that will stop [pubertal development] and might negatively impact the strength of your bones? According to me, there’s quite a difference between these two [ways of describing what medical decision-making competence entails]* (Focus group with clinicians)

#### Assessing MDC

Some clinicians stated that they assess MDC differently depending on the adolescent’s developmental age. They wondered what one can expect from an X-year-old child or adolescent with regard to, for example, understanding and appreciating what the treatment and its consequences entail. Some parents mention this too.

*That’s what I find difficult about medical decision-making competence: you verify whether someone has understood the information and to what extent someone can appreciate the consequences of the treatment in the future, but to what extent can an 11 year-old understand and appreciate that future properly? I think most 11 year-olds aren’t quite able to do that yet. But that doesn’t mean someone lacks decision-making competence, that’s simply appropriate for the [child’s] developmental age* (Focus group with clinicians)

*Of course we don’t know to what extent a child of that age can already understand and appreciate an entire lifetime. [...] That [not being able to understand and appreciate things when you haven't experienced them] is not only inherent to being a child [this is also true for adults], but when you are older you have seen a lot more of the world and you know what the impact can be on a person's life and a child doesn’t* (Interview with a parent of a transgirl who continued PS; age at start PS: 12.0; age at interview: 18.1)

Some parents mentioned that no matter how much information you receive prior to starting treatment, and no matter how much thought you put into this, there are some things that you simply cannot know or understand before you experience it.

*Some things you just don’t and can’t know until you’ve experienced it. Some things you need to experience before you know* (Interview with a transgirl who continued PS; age at start PS: 12.9; age at interview: 17.8)

In addition, some clinicians mentioned that sometimes an adolescent may not have MDC regarding a specific part or consequence of the treatment.

*In that case the adolescent doesn’t have medical decision-making competence regarding that aspect [the ability to decide about possible loss of fertility] [...] that [medical decision-making competence] develops much later in this area. […] And are you then going to postpone the start of treatment in the meantime [until the adolescent has medical decision-making competence regarding this aspect]? That's pretty complicated* (Focus group with clinicians)

Furthermore, the clinicians stated that in their daily practice, MDC is generally assessed implicitly and not in a structured way.

*We [as health care providers] haven’t quite formalized it [the assessment of medical decision-making competence] as some kind of medical decision-making competence measurement. Nevertheless, of course you do it [assess one’s medical decision-making competence]; you look at what exactly did the person tell me, what were his/her thoughts about it, and what can he/she tell me about the idea of how it [life after the start with puberty suppression] will go forward. And as a matter of fact, that also includes an assessment of the extent to which this person can understand and appreciate it [starting treatment with puberty suppression and its consequences]* (Focus group with clinicians)

### Aspects that are considered when assessing the adolescent’s MDC

The adolescents, their parents and clinicians described several aspects they take into account when assessing MDC to start PS. From the interviews and the focus groups, three subtopics emerged.

#### Understanding and appreciating what the treatment and its (long-term) consequences entail, and making the decision deliberately

The informants stated that understanding relevant information regarding the treatment is necessary for MDC. Nevertheless, only a few adolescents and their parents stated that the adolescent fully understood what PS entailed before starting the treatment. Some adolescents mentioned that they were able to understand most of what the treatment entailed.

*You receive so much explanation about it [the treatment with puberty suppression and its (possible) consequences] and you also have to fill in several forms. So you really know what it entails* (Interview with a transgirl who continued PS; age at start PS: 12.9; age at interview: 17.8)

*I don’t know if I understood it [what the treatment and its consequences entail] for 100 percent. But I knew that I could always stop it [the treatment] and that the male hormones would then come back. I knew that they were monthly injections, that they would stop [the release of] my male hormones in terms of physical aspects.. [like] hair growth or lowering of the voice. So in that respect, I was aware of what it [the treatment and its consequences] did and what it [the treatment and its consequences] was [were]* (Interview with a transgirl who continued PS; age at start PS: 12.4; age at interview: 18.5)

*I don’t think so [that my child made a deliberate decision to start puberty suppression], because we actually didn’t even know what the disadvantages [of the treatment] were* (Interview with a parent of a transgirl who continued PS; age at start PS: 12.4; age at interview: 18.6)

Three adolescents who proceeded to GAH after PS stated that during the time that they received treatment with PS, they began to better understand what the treatment involved.

*I think that I did take it in [the information about puberty suppression] back then [when I thought about starting the treatment with puberty suppression], but that I didn’t understand it very well. However, over the years, let’s say between 13 and 15 [or] 16 [years of age], I started to really understand the consequences that [the treatment] has, why I was doing it, why this was helping me, why it could also serve as extra time for reflection. Basically, everything to do with it [the treatment and its (possible) consequences]* (Interview with a transgirl who continued PS; age at start PS: 12.9; age at interview: 17.8)

*[I] maybe [understood] for about three quarters [what the treatment entailed before I started the treatment]. After the first injection [with puberty suppression] I was like, well I understand what they mean. And when I really noticed that nothing changed, I was like, I think I fully understand it now. So I understood most of it [what the treatment entailed before I started the treatment]* (Interview with a transboy who continued PS; age at start PS: 12.0; age at interview: 16.3)

Some clinicians wondered to what extent adolescents should be able to understand the information regarding the treatment to be decision-making competent.

*Are you [the adolescent] able to understand the information I provide? [The question then is] Where do you draw the line?* (Focus group with clinicians)

The informants stated that, besides being able to understand the relevant information about the treatment, adolescents need to be able to understand and appreciate the (long-term) consequences of the treatment to be decision-making competent. However, most adolescents and parents indicated that they/their children were not actually able to understand and appreciate all the (long-term) consequences.

*I was aware of all the disadvantages [of the treatment with puberty suppression]. Especially the mood swings, and I did underestimate those I think [the adolescent laughs]* (Interview with a transboy who continued PS; age at start PS: 10.9; age at interview: 17.6)

*She [my daughter] was well informed [about the treatment with puberty suppression and its (possible) consequences], she really understood, but neither we as parents nor X [my daughter] knew [beforehand] what it would be like* (Interview with a parent of a transgirl who continued PS; age at start PS: 12.9; age at interview: 17.8)

*When they are so small [younger than 12 years old] they [...] only have one goal in mind, namely: to become the woman you feel you are. I find it hard to assess whether she [my daughter] had really understood and appreciated that [the effects of the treatment and its (possible) consequences]. I don’t think that they [adolescents of that age] are able to understand and appreciate all of it* (Interview with a parent of a transgirl who continued PS; age at start PS: 12.0; age at interview: 18.1)

In addition to understanding the relevant information, and being able to understand and appreciate the (long-term) consequences, the informants wondered about how deliberate the adolescent’s decision to start with PS was. Clinicians stated that adolescents should be able to appreciate the impact of the treatment on their own situation. Of importance, most parents of adolescents who proceeded to GAH, as well as a few parents of adolescents who discontinued PS, thought that their child’s decision to start with PS was made deliberately. In contrast, most adolescents themselves, both continuers and discontinuers, thought they were not aware of the importance and impact of the decision.

*Of course, I was very young at the time [when I decided about starting the treatment with puberty suppression], but I had been whining about it for a long time already. It was more like: ‘I have to do it, I have to do it’. Did I think it through [what the treatment with puberty suppression and its (possible) consequences entailed]? No. Was I eventually satisfied with it [the treatment with puberty suppression]? Yes* (Interview with a transgirl who continued PS; age at start PS: 14.2; age at interview: 17.9)

*I think X [my daughter] understood that [what the treatment with puberty suppression and its (possible) consequences entailed] very well. [...] She did really make a deliberate decision [about whether or not to start treatment with puberty suppression]: ‘this is what I want’* (Interview with a parent of an assigned female at birth who had discontinued PS; age at start PS: 16.7; age at discontinuation PS: 17.0; age at interview: 19.5)

*Especially when it regards gender dysphoria, you [as a health care provider] want to hear from that person [who’s having gender incongruent feelings] what that person needs to feel good. And that doesn’t necessarily involve [a treatment with] testosterone and surgery and this and that. So then you need that person to be able to explain ‘well you know, this is what bothers me, and I don’t need this [kind of treatment]’. So you need to be able to have a kind of meaningful conversation about that* (Focus group with clinicians)

#### Reversibility of PS

The adolescents had diverging views regarding the way the fact that effects of PS are largely medical reversible influenced their decision-making process.

*I think that the fact that it [the effects of the treatment with puberty suppression] was [were] reversible lowered the threshold [to start the treatment]. And I think if it had immediately been about testosterone, then that doubt that subconsciously was already there, might have come to the surface, because that threshold [to start testosterone treatment] would have been higher, so I don’t know if I would still have started [the medical treatment] at that point. The fact that it [the effects of the treatment with puberty suppression] was [were] reversible definitely made it much easier for me to just think, yeah, I’m going to do this [start treatment with puberty suppression]* (Interview with an assigned female at birth who had discontinued PS; age at start PS: 16.7; age at discontinuation PS: 17.0; age at interview: 19.5)

*I didn’t care whether it [the effects of the treatment with puberty suppression] was [were] irreversible or not. [...] I didn’t really think about that at all. It was just something that I wanted so badly, one of my greatest wishes that would finally become true. So no, you don’t really think about that. I have had plenty of time beforehand [before I decided about starting the treatment] to think about it [the treatment and (possible) consequences]. They had already given me more than enough information. It was just something that felt right* (Interview with a transboy who continued PS; age at start PS: 12.0; age at interview: 16.3)

Some adolescents and clinicians thought that parents find it a reassuring idea that the first medical step has effects that are largely medically reversible. Some parents confirmed this idea whereas others did not.

*I think that especially for my parents, the decision to start treatment with puberty suppression was easier [compared to the decision to start treatment with gender affirming hormones]* (Interview with a transgirl who continued PS; age at start PS: 12.9; age at interview: 17.8)

*Especially for ourselves [as parents] it was extra time to reflect and think. I liked the idea that it [the effects of the treatment with puberty suppression] was [were] still reversible, even though I didn’t doubt her [gender incongruent] feelings or think that would ever be necessary. But I liked that about it [the treatment]. So I think the way we [as parents] experienced it [the fact that the effects of the treatment with puberty suppression are reversible] was different from the way she [our daughter] did; for her it was more like the beginning of [gender affirming medical] treatment* (Interview with a parent of a transgirl who continued PS; age at start PS: 12.0; age at interview: 18.1)

The clinicians had diverging views on the fact that effects of PS are largely medical reversible and the role this should play in the decision-making process.

*I also think, even though it [the effects of the treatment with puberty suppression] is [are] reversible, it is still an invasive treatment with substantial disadvantages* (Focus group with clinicians)

*You wonder if 11, 12, [and] 13 year-olds can really understand and appreciate what they are getting into [when starting treatment with puberty suppression]. But especially for ourselves, as psychologists, it is helpful that it [the effects of the treatment with puberty suppression] is [are] reversible* (Focus group with clinicians)

#### The role of age, intelligence, and mental health problems

The informants mentioned several factors they consider when assessing the adolescent’s MDC, among others, the adolescent’s developmental age, intelligence, and the presence of mental health problems. Clinicians stated that the younger the adolescent is when deciding about PS, the less likely to understand and appreciate what the treatment and its consequences entail.

*I also find very young children [...] aged 10 or 11 [...] tricky, the ones who don’t necessarily have a low IQ, but are just very young. And how they consider it [the treatment with puberty suppression and its (possible) consequences]* (Focus group with clinicians)

Adolescents and parents mentioned the role age plays when deciding about PS as well. Several adolescents stated that they thought they were not too young to decide about starting the treatment, but that as they grew older, their ability to make the decision improved.

*In that case [if I had made the decision to start treatment with puberty suppression when I was 16 or above] it would have been different. Then I would have had better abstract reasoning, better than when I was, say 13 years of age* (Interview with a transgirl who continued PS; age at start PS: 12.9; age at interview: 17.8)

*I don’t really think that I was too young to decide whether or not to start treatment with puberty suppression. Especially because that [the effects of the treatment] was [were] just reversible. Nevertheless, I do think that I was too young to completely understand it; the whole concept of transitioning, the social transition and the medical transition. Especially because I was only 14 [years of age] at the time, it was just like ‘this [treatment with puberty suppression] is the holy grail’. And only when I got older I grasped ‘hmm.. there is also another side to it [the treatment]* (Interview with an assigned female at birth who had discontinued PS; age at start PS: 16.7; age at discontinuation PS: 17.0; age at interview: 19.5)

In addition, clinicians mentioned that adolescents with lower intelligence might be less likely to be able to understand and appreciate what the treatment and its consequences entail. For several clinicians, low intelligence might even be a reason not to start PS, despite the presence of gender incongruent feelings. One adolescent and one parent mentioned the role of intelligence too. They stated that she/her child was smart enough to be able to understand and appreciate the consequences of PS prior to starting the treatment.

*I’m pretty smart so to say. So I could think of that [the effects of treatment and its (possible) consequences]* (Interview with an assigned female at birth who had discontinued PS; age at start PS: 12.1; age at discontinuation PS: 13.3; age at interview: 14.3)

*How smart they [the adolescents] are, is of course an important part of their competence to make medical decisions* (Focus group with clinicians)

*Especially those who have a disharmonic intelligence profile, who are verbally quite strong, but of whom you can wonder whether he/she is able to reason, and understand and appreciate the information [about the treatment and its (possible) consequences]. We do take more time for these cases [even though] we don’t doubt the diagnosis [of gender dysphoria]* (Focus group with clinicians)

Furthermore, clinicians mentioned that the presence of (serious) mental health problems and/or other developmental (like autism spectrum disorder) or physical differences (like deafness) might affect the adolescent’s MDC. Some wondered how MDC should be assessed in those circumstances.

*I think that, when you’re dealing with for example [someone with an] autism spectrum disorder, a deaf person, or someone with a very low intelligence, you have the idea that it almost becomes a black box; that you almost have to deduce the behavior [of the adolescent] to have an idea of what is happening inside that black box and how plausible is it that the adolescent has ‘authentic’ gender dysphoria?* (Focus group with clinicians)

### Relevance of MDC

Finally, one of the clinicians questioned why MDC to start PS is seen as such an important aspect to be eligible to start the treatment. The clinician wondered whether some people might assume that there is a direct correlation between MDC and the chance of having regrets about the decision to start the treatment later in life, even though competent adolescents who start PS may potentially still have regrets about this decision.

*Why do we insist on medical decision-making competence, if it were about regret [of the treatment], could we argue that if it [what the treatment with puberty suppression and its (possible) consequences entails] has been discussed, it has become some kind of a deliberate choice, which makes it less likely you will regret it?* (Focus group with clinicians)

This theme did not feature in any of the interviews with the adolescents or the parents.

## Discussion

Using qualitative methods, this study aimed to explicate and compare the perceptions of transgender adolescents who had continued or discontinued PS, their parents, and clinicians regarding adolescent’s MDC to start PS. From thematic analysis three themes emerged, being: challenges when assessing MDC to start PS, aspects that are considered when assessing the adolescent’s MDC, and relevance of MDC.

### Challenges when assessing MDC to start PS

Several aspects the participants mentioned illustrate ethical challenges surrounding assessing adolescents’ MDC to start PS. One of these aspects is the fact that certain consequences of PS and uncertainty about long-term effects cause doubts. Similar ethical challenges play a role in other fields. For example, in children with limited treatment options for serious conditions, ‘experimental’ interventions such as gene therapy may be seen as the best available option [45]. GnRH agonists are used as standard care for children with precocious puberty and an increasing number of other indications, and adverse psychological and physical effects have been rare [46–48]. Nevertheless, several adolescents, parents and clinicians in the current study share a feeling of unease regarding PS. They try to find a balance between the need to relieve the distress associated with the undesired endogenous pubertal development of the transgender adolescent, and the wish to avoid potential long-term negative effects of PS [49]. This is difficult since what the best care is, depends also on individual preferences. Even though more evidence-based outcomes of treatment is important, it remains impossible to predict the treatment’s effects and impact on a particular individual.

One of the consequences mentioned by the participants was the possible loss of fertility. Interestingly, several adolescents, most of whom were continuers, parents, and all clinicians had a specific feeling of unease about this. One could therefore question to what extent or in what way potential loss of fertility should already be taken into account when assessing adolescents’ MDC to start PS.

Although the effects of PS on the development of secondary sex characteristics and gonadal function are reversible when the treatment is discontinued, as far as is currently known, if adolescents subsequently undergo treatment with GAH and gonadectomy, this will result in loss of fertility [2]. If they start PS at a young age, they may never undergo their endogenous puberty and may therefore not be able to pursue fertility preservation [14, 15]. However, not all adolescents pursue gonadectomy, and depending on birth-assigned sex and the type of treatment individuals choose to undergo, fertility outcomes may vary [50]. Research shows that very few (1.9–6%) adolescents discontinue PS [e.g. 12, 51, 52]. A subset of these adolescents (3.5–3.7%) no longer wish gender-affirming treatment [12, 51]. That means that the vast majority of the adolescents who start PS subsequently proceed to GAH, with possible loss of fertility as a result. Providing adequate information about the impact of treatment on future fertility and about fertility preservation is therefore highly recommended [53, 54].

However, the adolescents, parents and clinicians wondered to what extent an adolescent should and can be able to understand and appreciate some possible (long-term) consequences of the treatment. According to clinicians and parents, not being able to understand and appreciate the impact of the loss of fertility on one’s future life and relationships is inherent to an adolescent’s age and developmental stage. Some clinicians mentioned that even some adults are not able to understand and appreciate the impact of certain consequences of the treatment. Thus, one might question whether it is reasonable to expect adolescents to be able to understand and appreciate all possible consequences of medical treatment. Yet we often seem to assess adults’ MDC in these situations as ‘sufficient’, while we question that of adolescents. Infertility and concerns about (future) fertility may have a major negative impact on quality of life and mental health [55, 56]. Research focusing on survivors of pediatric cancer shows that plans of youth for future children may change over the years [53, 54]. Although there is, as far as we know, no similar published data regarding transgender youth deciding on PS, data from a Dutch study with transgender adults shows that views regarding parenthood might change over time [57]. Other research shows that vitality and self-perceived mental health status is significantly better among adult transmen with children than for those without [58]. Offering the possibility of fertility preservation is therefore important. However, that in turn might bring its own difficulties. For example, the fertility preservation process might have a psychological impact on the adolescent and/or the parent(s), as mentioned by one of the parents in this study. In our clinical practice, all adolescents and their families are offered fertility counseling, but some families refuse because they consider even just the counseling too psychologically burdensome for their young child.

Up until now little is known about the possible psychological impact of the procedures and the process of fertility preservation on transgender adolescents [59]. Further research regarding not only the benefit but also the possible harm of a fertility preservation process on transgender adolescents and their families would therefore be valuable [59, 60]. Besides, we recommend future research examining the impact of the loss of fertility later in the adolescents’ lives, and investigating the best way to communicate information regarding fertility (preservation) to these young adolescents. Of note, in the Netherlands fertility preservation continues to be a topic of conversation throughout the diagnostic and treatment phases, and in any case before decisions about GAH and surgery are made, since fertility preservation remains possible even after adolescents have started treatment with PS, for example, by temporarily interrupting treatment.

Another aspect participants mentioned regarding assessing MDC to start PS was the parents’ role. Involvement and help of parents with regard to making medical decisions for minors is important, just as most adolescents, both continuers and discontinuers, parents, and clinicians mentioned, and as stated in laws and in international guidelines on transgender care for children and adolescents [1, 5]. Additionally, the clinicians in this study said that, besides the adolescents and parents, they themselves took part in the decision-making process too. None of the adolescents and parents did not mention this. For patient-centered care shared decision-making (SDM) is considered essential and it is recommended by pediatric regulatory organizations [61, 62]. The SDM approach in general care is evidence-based and promotes collaboration between patients, family members, and clinicians when making a decision regarding health care [63]. Patients, family members, and clinicians can deliberately decide about the best treatment plan by exchanging information about the treatment’s evidence (options, benefits, and risks) and the patient’s and family’s preferences [64]. In SDM the patient’s expertise and values are considered along with empirical medical information, and the decision-making responsibilities of the patient, family members, and clinicians are balanced [65–67].

Although little is known about SDM in the context of PS for transgender adolescents, research shows that SDM can support decisions about GAH treatment in transgender adolescents when integrating into practice the following five conditions: open communication, role agreement, supportive relationships, agreement about the decision, and sufficient time [68]. Research shows that among other things the use of information-sharing techniques that are age-appropriate, breaking down a decision into smaller choices, and asking direct and simple questions all promote adolescents’ ability to participate in medical discussion [69]. Future research examining how transgender adolescents can best be involved in the decision-making process regarding PS is recommended.

Additionally, it is notable that most adolescents who proceeded to GAH after PS, and parents mentioned that they did not feel they had a choice whether or not to start the treatment with PS. By contrast, none of the adolescents who discontinued PS or their parents explicitly stated having no choice. It is noteworthy that, most adolescents, continuers and discontinuers, and their parents mentioned not really taking the treatment’s possible negative consequences into consideration. This, even though most adolescents, parents, and clinicians stated that understanding the treatment and its consequences should be considered when assessing adolescents’ MDC. Apparently, the possible negative consequences of the treatment do not outweigh the burden of the adolescents’ gender incongruent feelings. One could therefore question whether adolescents’ MDC to start PS is at all ‘required’, when the adolescents might not even have a choice to make. This situation is not unique for the transgender adolescent care. For example, some patients undergoing deep brain stimulation do not have other treatment options left. Nevertheless, in the current medical model in the Netherlands, these patients still need to give their fully informed consent to the treatment [70]. In addition, it should be mentioned that one’s feeling of not having a choice is different from having no choice; in fact, adolescents still have a choice to proceed to treatment or not, but for them one option is significantly preferable (i.e. to receive treatment).

Besides, the adolescents, both continuers and discontinuers, parents, and clinicians questioned what the term ‘understanding’ means regarding information about the treatment and its possible consequences in the context of adolescents’ MDC to start PS; to what extent should an adolescent be able to understand the information regarding PS to be decision-making competent? Furthermore, most clinicians experienced challenges while assessing MDC and mentioned that they apply their own definition of MDC depending on the characteristics of the adolescent at hand. In addition, the results show MDC is generally assessed implicitly and not in a structured way in daily practice. This is in line with what earlier research in other contexts shows [40]. Except for one Dutch quantitative study, which shows that the vast majority (89%) of the examined transgender adolescents (aged 10–18 years) about to start PS treatment are competent to consent to this treatment, there is little evidence on transgender adolescents’ MDC to start PS [41]. Because clinicians indicate that they find it difficult to determine MDC, it would be desirable to develop a more uniform way to assess MDC and provide ethics support for the ethical dilemmas that are encountered when assessing MDC [71–73]. Dissemination of knowledge about MDC to start PS would help to adequately support adolescents, parents, and clinicians in the decision-making process. Despite the fact that current transgender clinical guidelines state that adolescent’s MDC is a prerequisite to start PS, the guidelines hardly clarify what ‘adolescents having MDC’ means in practice. Dutch researchers have, largely based on the information gained in the current study, developed an ethics support tool (in Dutch: ‘wilsbekwaamheidswijzer’) that provides clinicians information and direction on how to deal with adolescents’ MDC. The tool provides clinical guidance on assessing adolescents’ MDC, for example regarding what aspects the adolescents should understand about the treatment before they are considered competent [74, 75]. Making such an ethics support tool available to clinicians in other countries as well could be very helpful. Additionally, clinicians working in transgender treatment teams in the Netherlands rated Moral Case Deliberation, a relatively well-established form of clinical ethics support, as highly valuable in dealing with moral challenges in their clinical practice [74, 76]. Moral Case Deliberation could also be used by the transgender treatment teams when, in clinical practice, they are confronted with moral challenges regarding adolescents’ MDC to start PS and/or its assessment.

### Aspects that are considered when assessing the adolescent’s MDC

Results further showed that the adolescents, parents, and clinicians mentioned several (contextual) aspects that, according to them, should be considered when assessing the adolescent’s MDC to start PS. One aspect various adolescents, parents, and clinicians mentioned with regard to this, was the understanding of the treatment and its consequences. Various adolescents, both continuers and discontinuers, mentioned that before they started PS, they were not aware of some of the psychosocial consequences of delaying puberty while their peers underwent multifaceted developmental accomplishments. An example of a potentially negative consequence of keeping the adolescent in a prepubertal state is isolating the adolescent from peers [77]. On the other hand, research shows that the adolescents’ psychological functioning improved or did not change after starting specialized transgender care involving PS [78–80]. Only a few of the adolescents and their parents stated that they fully understood what PS and its consequences entailed, but even so, the adolescents found themselves able to decide about whether or not to start the treatment. In both the beforementioned quantitative study regarding the assessment of MDC in transgender adolescents and the current qualitative study, the adolescents are judged competent and find themselves competent to decide about starting PS [41]. Seemingly, fully understanding and appreciating the treatment are not requirements for MDC to start PS. This is in line with the statements of some clinicians and parents that not being able to understand and appreciate the impact of certain consequences of PS is inherent to the age, developmental stage and/or life experience of the adolescent, just as previous research in other contexts has shown [40].

Age was another factor that most participants mentioned that may have a decisive impact on MDC, and should therefore be considered when assessing the adolescents’ MDC. Age is often considered to be the best indicator of MDC [81]. Research shows that children aged ≥ 12 years may have MDC, provided they have favorable environmental factors [82, 83]. On the other hand, the same research shows that there is no universal agreement regarding the age at which children can reasonably be expected to have MDC regarding every decision in every context. Early development of the reward system of the brain in combination with late development of the control system reduces adolescents’ MDC in certain challenging contexts which are not supportive [82]. Therefore, children and adolescents of the same age may have different levels of maturity and there is no general clear cut-off at which all children or adolescents have MDC [82]. Furthermore, some experts argue that children who have personal experiences with ‘illness’, may have greater understanding and insight compared to children who do not have this experience [84–86]. This may specifically play a role in the case of gender nonconforming youth, where most adolescents seen at a gender identity clinic have long lasting or even life-long gender incongruent feelings. However, research does not confirm this hypothesis [41, 81]. It is worthwhile considering to assess MDC and maturity on an individual basis rather than using a fixed age criterium, although the fact that most participants in the current study experienced age as an important aspect concerning MDC, may support that for certain more irreversible components of gender-affirming treatment (like treatment with GAH and surgeries), age criteria should remain existing [1, 87, 88].

### Relevance of MDC

Finally, in our study clinicians pondered whether too much importance is placed on the adolescent’s MDC. None of the adolescents and parents did mention this. So far, there is no direct correlation between having MDC and not having regrets about a decision later in life, something that some stakeholders seem to have in mind [26]. Besides, respecting an individual’s autonomy encompasses one’s right to make a decision that is regretted later on in life [89]. A balance that needs to be struck is between the risk of regret and the risk of not providing the treatment, since refraining from treatment might have harmful effects too [17, 26].

### Strengths and weaknesses

There are some strengths and weaknesses to the present study. The qualitative nature of this study made it possible to find out, in depth, the ways in which transgender adolescents, their parents, and clinicians think about transgender adolescents’ MDC to start treatment with PS. Another strength of this study is that adolescents who did continue with GAH after PS as well as adolescents who did not proceed to GAH were interviewed. This allowed us to compare their considerations. Nevertheless, the retrospective nature of this study raises the possibility of recall bias and hindsight bias of the informants. In addition, the informants were recruited from two Dutch treatment teams using the same protocol prescribing that PS was required for all adolescents before any further affirming treatment was provided. Adolescents recruited from other treatment clinics in other contexts might report different considerations regarding MDC [27].

Therefore, we encourage prospective gathering of more qualitative data from adolescents who haven’t started PS yet, or receive PS but have not started treatment with GAH yet. Due to the small sample size, the nonparticipation rate and the skewed sex ratio, it is not completely certain if genuine saturation was reached. Nonparticipating adolescents who had discontinued treatment might have had other thoughts regarding the adolescents’ MDC compared to the adolescents that have been interviewed. We, therefore, encourage gathering more qualitative data from a larger sample with a more balanced sex ratio.

## Conclusion

In conclusion, this study shows that adolescents, their parents, and clinicians take various aspects into account regarding the adolescent’s MDC. The four criteria one needs to fulfill to have MDC - understanding, appreciating, reasoning, and communicating a choice - were all, to a greater or lesser extent, mentioned as challenging by the participants, just as MDC being relative to a specific decision and context [30]. Most adolescents, parents, and clinicians find understanding and appreciating what the treatment and its consequences entail, important for MDC. Nevertheless, even though most adolescents, both continuers and discontinuers, and parents felt they did not have a full understanding and appreciation of all consequences, they thought that they were able to make the decision to start PS. Parents’ support of their child was considered essential in the decision-making process. However, several parents and clinicians wondered to what extent they themselves, and adults in general, are able to understand and appreciate certain consequences, let alone adolescents. The results of the current study show that clinicians find MDC challenging to assess in a uniform way. Dissemination of knowledge and support concerning the assessment of MDC and encountered ethical dilemmas about transgender adolescents’ MDC is desirable in order for clinicians to support adolescents and parents in the decision-making process.

## Appendix A. Initial interview questions

1. For you, was the treatment to suppress puberty time to consider and decide on irreversible treatment or was the treatment important to you for other reasons?

2. How consciously did you make the decision to start PS at the time?

3. Have there been any consequences, or has there been any impact of the treatment with PS that you hadn’t foreseen, that you were not aware of, that were better or worse than you had expected?

4. To what extent did you make your decision to start PS in a different way from your decision to start with treatment with gender affirming hormones?

5. Is there anything that hasn’t been discussed in this interview but that you think is relevant in relation to the topic, anything that you would like to add?

## Footnotes

1 According to the WPATH Standards of Care and the Endocrine Society Guideline, transgender adolescents undergo a diagnostic trajectory with a mental health care provider over a longer period of time in order to assess eligibility for PS, discusses the benefits and potential risks of treatment, and be able to make a shared decision in a multidisciplinary team (adolescent, parents, health care providers).

2 According to the protocol as used in the Dutch gender identity clinics the upper limit of the person’s body mass index (BMI) to be eligible for for example mastectomy is 35 kg/m^2^, and for phalloplasty and vaginoplasty the upper limit is a BMI of 30 kg/m^2^.

## References

[1] Coleman E, Bockting WO, Botzer M, Cohen-Kettenis PT, DeCuypere G, Feldman J, Fraser L, Green J, Knudson G, Meyer WJ, Monstrey S, Adler RK, Brown GR, Devor AH, Ehrbar R, Ettner R, Eyler E, Garofalo R, Karasic DH (2012). Standards of care for the health of transsexual, transgender, and gender-nonconforming people, version 7. Int J Transgend.

[2] Hembree WC, Cohen-Kettenis PT, Gooren L, Hannema SE, Meyer WJ, Murad MH, Rosenthal SM, Safer JD, Tangpricha V, T’Sjoen GG (2017). Endocrine treatment of gender-dysphoric/gender-incongruent persons: an endocrine society clinical practice guideline. J Cain Endocrinol Metab.

[3] Vrouenraets LJJJ, de Vries MC, Hein IM, Arnoldussen M, Hannema SE, de Vries ALC (2021). Perceptions on the function of puberty suppression of transgender adolescents who continued or discontinued treatment, their parents, and clinicians. Int J Transgend Health.

[4] van de Grift TC, van Gelder ZJ, Mullender MG, Steensma TD, de Vries ALC, Bouman M (2020). Timing of puberty suppression and surgical options for transgender youth. Pediatrics.

[5] Vrouenraets LJJJ, Fredriks AM, Hannema SE, Cohen-Kettenis PT, de Vries MC (2015). Early medical treatment of children and adolescents with gender dysphoria: an empirical ethical study. J Adolesc Health.

[6] Laidlaw MK, van Meter QL, Hruz PW, van Mol A, Malone WJ (2019). Letter to the editor: “Endocrine treatment of gender-dysphoric/gender-incongruent persons: an endocrine society clinical practice guideline”. J Clin Endocrinol Metab.

[7] Chen D, Strang JF, Kolbuck VD, Rosenthal SM, Wallen K, Waber DP (2020). Consensus parameter: research methodologies to evaluate neurodevelopmental effects of pubertal suppression in transgender youth. Transgender Health.

[8] Vrouenraets LJJJ, Fredriks AM, Hannema SE, Cohen-Kettenis PT, de Vries MC (2016). Perceptions of sex, gender, and puberty suppression: a qualitative analysis of transgender youth. Arch Sex Behav.

[9] Dhejne C, Lichtenstein P, Boman M, Johansson AL, Långström N, Landén M (2011). Long-term follow-up of transsexual persons undergoing sex reassignment surgery: cohort study in Sweden. PLoS ONE.

[10] Wiepjes CM, den Heijer M, Bremmer MA, Nota NM, de Blok CJ, Coumou BJ, Steensma TD (2020). Trends in suicide death risk in transgender people: results from the Amsterdam cohort of gender dysphoria study (1972–2017). Acta Psychiatr Scand.

[11] Poštuvan V, Podlogar T, Šedivy NZ, Leo DL (2019). Suicidal behaviour among sexual-minority youth: a review of the role of acceptance and support. Lancet Child Adolesc Health.

[12] Brik T, Vrouenraets LJJJ, de Vries MC, Hannema SE (2020). Trajectories of adolescents treated with gonadotropin-releasing hormone analogues for gender dysphoria. Arch Sex Behav.

[13] Arnoldussen M, Steensma TD, Popma A, van der Miesen AIR, Twisk JW, de Vries ALC (2020). Re-evaluation of the Dutch approach: are recently referred transgender youth different compared to earlier referrals?. Eur Child Adolesc Psychiatry.

[14] Brik T, Vrouenraets LJJJ, Schagen SEE, Meissner A, de Vries MC, Hannema SE (2019). Use of fertility preservation among a cohort of transgirls in the Netherlands. J Adolesc Health.

[15] Hudson J, Nahata L, Dietz E, Quinn GP (2018). Fertility counseling for transgender AYAs. Clin Pract Pediatr Psychol.

[16] The Lancet child & adolescent health (2021). A flawed agenda for trans youth. Lancet Child Adolesc Health.

[17] de Vries ALC, Richards C, Tishelman AC, Motmans J, Hannema SE, Green J, Rosenthal SM (2021). Bell v Tavistock and Portman NHS foundation trust [2020] EWHC 3274: weighing current knowledge and uncertainties in decisions about gender-related treatment for transgender adolescents. Int J Transgend Health.

[18] Giordano S (2008). Lives in a chiaroscuro. Should we suspend the puberty of children with gender identity disorder?. J Med Ethics.

[19] Giordano S, Holm S (2020). Is puberty delaying treatment ‘experimental treatment’?. Int J Transgend Health.

[20] Kreukels BP, Cohen-Kettenis PT (2011). Puberty suppression in gender identity disorder: the Amsterdam experience. Nat Rev Endocrinol.

[21] Byne W, Bradley SJ, Coleman E, Eyler AE, Green R, Menvielle EJ, Meyer-Bahlburg HF, Pleak RR, Tompkins DA (2012). Report of the American psychiatric association task force on treatment of gender identity disorder. Arch Sex Behav.

[22] Levine SB (2019). Informed consent for transgendered patients. J Sex Marital Ther.

[23] d’Abrera JC, D’Angelo R, Halasz G, Prager S, Morris P (2020). Informed consent and childhood gender dysphoria: emerging complexities in diagnosis and treatment. Australas Psychiatry.

[24] Baron T, Dierckxsens G (2021). Two dilemmas for medical ethics in the treatment of gender dysphoria in youth. J Med Ethics.

[25] Giordano S, Garland F, Holm S (2021). Gender dysphoria in adolescents: can adolescents or parents give valid consent to puberty blockers?. J Med Ethics.

[26] Pang KC, Giordano S, Sood N, Skinner SR (2021). Regret, informed decision making, and respect for autonomy of trans young people. Lancet Child Adolesc Health.

[27] Levine SB, Abbruzzese E, Mason JW (2022). Reconsidering informed consent for trans-identified children, adolescents, and young adults. J Sex Marital Ther.

[28] Kerman HM, Pham A, Crouch JM, Albertson K, Salehi P, Inwards-Breland DJ, Ahrens KR (2021). Gender diverse youth on fertility and future family: a qualitative analysis. J Adolesc Health.

[29] Grisso T, Appelbaum PS, Hill-Fotouhi C (1997). The MacCAT-T: a clinical tool to assess patients’ capacities to make treatment decisions. Psychiatr Serv.

[30] Appelbaum PS, Grisso T (1988). Assessing patients’ capacities to consent to treatment. N Engl J Med.

[31] Grisso T, Appelbaum PS (1995). Comparison of standards for assessing patients’ capacities to make treatment decisions. Am J Psychiatry.

[32] Beauchamp TL, Childress JF (2008). Principles of Biomedical Ethics.

[33] Dyer C (2020). Children are “highly unlikely” to be able to consent to taking puberty blockers, rules high court. BMJ.

[34] Dyer C (2020). Puberty blockers: children under 16 should not be referred without court order, says NHS England. BMJ.

[35] Thornton J (2021). Court upholds Gillick competence in puberty blockers case. The Lancet.

[36] Naiingolan L (2021) Hormonal Tx of youth with gender dysphoria stops in Sweden. Medscape Medical News https://www.medscape.com/viewarticle/950964 Accessed 12 May 2021

[37] Armitage R (2021). The communication of evidence to inform trans youth health care. Lancet Child Adolesc Health.

[38] Moreton KL (2021). A backwards-step for Gillick: trans children’s inability to consent to treatment for gender dysphoria—quincy bell & Mrs A v the tavistock and portman NHS foundation trust and Ors [2020] EWHC 3274 (admin). Med Law Rev.

[39] Wheeler R (2021). Gillick’s third facet: where the child and the doctor agree on treatment of gender dysphoria. Arch Dis Child.

[40] Hein IM, Troost PW, Broersma A, de Vries MC, Daams JG, Lindauer RJL (2015). Why is it hard to make progress in assessing children’s decision-making competence?. BMC Med Ethics.

[41] Vrouenraets LJJJ, de Vries ALC, de Vries MC, der Miesen AIR, Hein IM (2021). Assessing medical decision-making competence in transgender youth. Pediatrics.

[42] American Psychiatric Association (2013). Diagnostic and statistical manual of mental disorders: DSM-5.

[43] Miles MB, Huberman AM (1994). Qualitative data analysis. An expanded sourcebook.

[44] Stake RE, Denzin NK, Lincoln YS (2005). Qualitative case studies. The sage handbook of qualitative research.

[45] Lyer AA, Saade D, Bharucha-Goebel D, Foley AR, Averion GM, Paredes E, Gray S, Bönnemann CG, Grady C, Hendriks S, Rid A (2021). Ethical challenges for a new generation of early-phase pediatric gene therapy trials. Genet Med.

[46] Krishna KB, Fuqua JS, Rogol AD, Klein KO, Popovic J, Houk CP, Charmandari E, Lee PA (2019). Use of gonadotropin-releasing hormone analogs in children: update by an international consortium. Horm Res Paediatr.

[47] Lee JW, Kim HJ, Choe YM, Kang HS, Kim SI, Jun YH, Lee JE (2014). Significant adverse reactions to long-acting gonadotropin-releasing hormone agonists for the treatment of central precocious puberty and early onset puberty. Ann Pediatr Endocrinol Metab.

[48] Yu R, Yang S, Hwang T (2019). Psychological effects of gonadotropin-releasing hormone agonist treatment in girls with central precocious puberty. J Pediatr Endocrinol Metab.

[49] Butler G, Wren B, Carmichael P (2019). Puberty blocking in gender dysphoria: suitable for all?. Arch Dis Child.

[50] Cheng PJ, Pastuszak AW, Myers JB, Goodwin IA, Hotaling JM (2019). Fertility concerns of the transgender patient. Transl Androl Urol.

[51] Khatchadourian K, Amed S, Metzger DL (2014). Clinical management of youth with gender dysphoria in vancouver. J Pediatr.

[52] Wiepjes CM, Nota NM, de Blok CJ, Klaver M, de Vries ALC, Wensing-Kruger SA, de Jongh RT, Bouman M, Steensma TD, Cohen-Kettenis PT, Gooren LJG, Kreukels BPC, den Heijer M (2018). The Amsterdam cohort of gender dysphoria study (1972–2015): trends in prevalence, treatment, and regrets. J Sex Med.

[53] Armuand GM, Wettergren L, Rodriguez-Wallberg KA, Lampic C (2014). Desire for children, difficulties achieving a pregnancy, and infertility distress 3 to 7 years after cancer diagnosis. Support Care Cancer.

[54] Stein DM, Victorson DE, Choy JT, Waimey KE, Pearman TP, Smith K, Dreyfuss J, Kinahan KE, Sadhwani D, Woodruff TK, Brannigan RE (2014). Fertility preservation preferences and perspectives among adult male survivors of pediatric cancer and their parents. J Adolesc Young Adult Oncol.

[55] Carter J, Raviv L, Applegarth L, Ford JS, Josephs L, Grill E, Sklar C, Sonoda Y, Baser RE, Barakat RR (2010). A cross-sectional study of the psychosexual impact of cancer-related infertility in women: third-party reproductive assistance. J Cancer Surviv.

[56] Trent ME, Rich M, Austin B, Gordon C (2003). Fertility concerns and sexual behaviour in adolescent girls with polycystic ovary syndrome: Implications for quality of life. J Pediatr Adolesc Gynecol.

[57] Van Mello N, De Nie I, Asseler J, Arnoldussen M, Steensma T, Den Heijer M, De Vries A, Huirne J (2022). P-506 reflecting on the importance of family building and fertility preservation: transgender people’s experiences with starting gender-affirming treatment as adolescent. Hum Reprod.

[58] Wierckx K, van Caenegem E, Pennings EG, Elaut E, Dedecker D, van de Peer F, Steven Weyers S, de Sutter P, T'Sjoen G (2012). Reproductive wish in transsexual men. Hum Reprod.

[59] Baram S, Myers SA, Yee S, Librach CL (2019). Fertility preservation for transgender adolescents and young adults: a systematic review. Hum Reprod Update.

[60] Chen D, Simons L (2018). Ethical considerations in fertility preservation for transgender youth: a case illustration. Clin Pract Pediatr Psychol.

[61] Barry MJ, Edgman-Levitan S (2012). Shared decision making-the pinnacle patient-centered care. N Engl J Med.

[62] Harrison C, Canadian Paediatric Society (CPS), Bioethics Committee (2004). Treatment decisions regarding infants, children and adolescents. Paediatr Child Health.

[63] Boland L, Graham ID, Légaré F, Lewis K, Jull J, Shephard A, Lawson ML, Davis S, Yameogo A, Stacey D (2019). Barriers and facilitators of pediatric shared decision-making: a systematic review. Implement Sci.

[64] Légaré F, Stacey D, Gagnon S, Dunn S, Pluye P, Frosch D, Kryworuchko J, Elwyn G, Gagnon M, Graham ID (2011). Validating a conceptual model for an inter-professional approach to shared decision making: a mixed methods study. J Eval Clin Pract.

[65] Makoul G, Clayman ML (2006). An integrative model of shared decision making in medical encounters. Patient Educ Couns.

[66] Crickard EL, O’Brien MS, Rapp CA, Holmes CL (2010). Developing a framework to support shared decision making for youth mental health medication treatment. Community Ment Health J.

[67] Langer DA, Jensen-Doss A (2018). Shared decision-making in youth mental health care: using the evidence to plan treatments collaboratively. J Clin Child Adolesc Psychol.

[68] Clark BA, Virani A, Marshall SK, Saewyc EM (2021). Conditions for shared decision making in the care of transgender youth in Canada. Health Promot Int.

[69] Michaud PA, Blum RW, Benaroyo L, Zermatten J, Baltag V (2015). Assessing an adolescent’s capacity for autonomous decision-making in clinical care?. J Adolesc Health.

[70] Schermer M (2011). Ethical issues in deep brain stimulation. Front Integr Neurosci.

[71] Hartman LA, Metselaar S, Molewijk BAC, Edelbroek HM, Widdershoven GA (2018). Developing an ethics support tool for dealing with dilemmas around client autonomy based on moral case deliberations. BMC Med Ethics.

[72] Hartman LA, Widdershoven GA, de Vries ALC, Wensing-Kruger SA, den Heijer M, Steensma TD, Molewijk BAC (2019). Integrative clinical ethics support in gender affirmative care: lessons learned. HEC Forum.

[73] Hein IM, Hondius AJK (2017). Wilsbekwaamheid in de medische praktijk.

[74] Molewijk BAC, Abma T, Stolper M, Widdershoven GA (2008). Teaching ethics in the clinic. The theory and practice of moral case deliberation. J Med Ethics.

[75] De Snoo-Trimp JC, de Vries ALC, Molewijk BAC, Hein IM How to deal with moral challenges around the decision-making competence in transgender adolescent care? Development of an ethics support tool. Manuscript submitted for publication 10.1186/s12910-022-00837-1 PMC9494804 36138384

[76] Vrouenraets LJJJ, Hartman LA, Hein IM, de Vries ALC, de Vries MC, Molewijk BAC (2020). Dealing with moral challenges in treatment of transgender children and adolescents: evaluating the role of moral case deliberation. Arch sex behav.

[77] Rosenthal SM (2014). Approach to the patient: transgender youth: endocrine considerations. J Clin Endocrinol Metab.

[78] van der Miesen AIR, Steensma TD, de Vries ALC, Bos H, Popma A (2020). Psychological functioning in transgender adolescents before and after gender-affirmative care compared with cisgender general population peers. J Adolesc Health.

[79] Carmichael P, Butler G, Masic U, Cole TJ, De Stavola BL, Davidson S, Skageberg EM, Khadr S, Viner RM (2021). Short-term outcomes of pubertal suppression in a selected cohort of 12 to 15 year old young people with persistent gender dysphoria in the UK. PLoS ONE.

[80] Rew L, Young CC, Monge M, Bogucka R (2021). Puberty blockers for transgender and gender diverse youth - a critical review of the literature. Child Adolesc Ment Health.

[81] Hein IM, Troost PW, Lindeboom R, Benninga MA, Zwaan CM, van Goudoever JB, Lindauer RJL (2015). Key factors in children’s competence to consent to clinical research. BMC Med Ethics.

[82] Grootens-Wiegers P, Hein IM, van den Broek JM, de Vries MC (2017). Medical decision-making in children and adolescents: developmental and neuroscientific aspects. BMC Pediatr.

[83] Hein IM, Troost PW, Lindeboom R, Benninga MA, Zwaan CM, van Goudoever JB, Lindauer RJL (2014). Accuracy of the MacArthur competence assessment tool for clinical research (MacCAT-CR) for measuring children’s competence to consent to clinical research. JAMA Pediatr.

[84] Alderson P (2007). Competent children? Minors’ consent to health care treatment and research. Soc Sci Med.

[85] Bluebond-Langner M, Belasco JB, DeMesquita WM (2010). “I want to live, until I don’t want to live anymore”: involving children with life-threatening and life-shortening illnesses in decision making about care and treatment. Nurs Clin North Am.

[86] Larcher V, Hutchinson A (2010). How should paediatricians assess Gillick competence?. Arch Dis Child.

[87] Hein IM, De Vries MC, Troost PW, Meynen G, Van Goudoever JB, Lindauer RJL (2015). Informed consent instead of assent is appropriate in children from the age of twelve: policy implications of new findings on children’s competence to consent to clinical research. BMC Med Ethics.

[88] Proposed version of the Standards of Care version 8 (SOC8) (2021) Chapter Adolescent. https://www.wpath.org/media/cms/Documents/SOC%20v8/SOC8%20Chapters%20for%20Public%20Comment/SOC8%20Chapter%20Draft%20for%20Public%20Comment%20-%20Adolescent.pdf?_t=1638731433

[89] Glover J (1990). Causing death and saving lives: The moral problems of abortion, infanticide, suicide, euthanasia, capital punishment, war and other life-or-death choices.

